# A Real-Time Recombinase Polymerase Amplification Method for Rapid Detection of *Vibrio vulnificus* in Seafood

**DOI:** 10.3389/fmicb.2020.586981

**Published:** 2020-11-06

**Authors:** Xiaohan Yang, Xue Zhang, Yu Wang, Hui Shen, Ge Jiang, Jingquan Dong, Panpan Zhao, Song Gao

**Affiliations:** ^1^Jiangsu Key Laboratory of Marine Biological Resources and Environment, Jiangsu Key Laboratory of Marine Pharmaceutical Compound Screening, Co-Innovation Center of Jiangsu Marine Bio-industry Technology, School of Pharmacy, Jiangsu Ocean University, Lianyungang, China; ^2^Jiangsu Institute of Oceanology and Marine Fisheries, Nantong, China; ^3^Key Laboratory of Zoonosis Research by Ministry of Education, College of Veterinary Medicine, Jilin University, Changchun, China

**Keywords:** *Vibrio vulnificus*, real-time recombinase polymerase amplification, rapid detection, recombinase polymerase amplification, extracellular metalloprotease

## Abstract

As an important foodborne pathogen, *Vibrio vulnificus* gives a significant threat to food safety and public health. Rapid and accurate detection methods for *V. vulnificus* are required to control its spread. The conventional detection methods are time-consuming and labor-intensive, while the polymerase chain reaction (PCR)- and quantitative PCR (qPCR)-based methods are limited because of their dependence on laboratory equipment. Nucleic acid isothermal amplification technologies have been applied to develop simpler assays. In this study, a rapid detection method based on real-time recombinase polymerase amplification (RPA) targeting the extracellular metalloprotease (*empV*) gene of *V. vulnificus* has been established. The method finished the detection in 2–14 min at 39°C with good specificity. The limit of detection was 17 gene copies or 1 colony-forming unit (CFU) per reaction, or 1 CFU/10 g of spiked food with enrichment. In a clinical sample detection test, the results of real-time RPA were 100% consistent with bioassay and qPCR. Moreover, the method could resist the effect of food matrix and could tolerate crude templates. The real-time RPA method established in this study is rapid and simple and has the potential to be widely applied for *V. vulnificus* detection in food safety control.

## Introduction

Foodborne infectious diseases are of significant public health concern. Researchers have focused on the development of rapid and reliable methods of pathogen detection for food safety. *Vibrio vulnificus* is a Gram-negative, halophilic bacterium found in coastal or estuarine environment worldwide and has been isolated from sediments, water, and a variety of seafood (Dalsgaard et al., 1996; Jones and Oliver, 2009). *V. vulnificus* is regarded as one of the major causes of seafood-associated diseases. Consumption of raw or undercooked seafood contaminated with *V. vulnificus* can result in severe infection that causes life-threatening septicemia and acute gastroenteritis (Jones and Oliver, 2009). Wound infections can also occur when one is exposed to contaminated sea water or seafood (Hlady and Klontz, 1996; Strom and Paranjpye, 2000). The mortality rate of *V. vulnificus* infection is up to 60%, making it a serious public health and food safety concern (Jones and Oliver, 2009). With the global warming, this bacterium inhabits a warm environment and causes outbreaks in more areas (Rippey, 1994; Huehn et al., 2014; Bonnin-Jusserand et al., 2019; Leng et al., 2019). Thus, rapid, specific, and reliable detection methods for *V. vulnificus* are particularly required to facilitate better control of its spread.

The conventional methods for the detection of *V. vulnificus* usually include pre-enrichment, isolation, and biochemical identifications, which are time-consuming and labor-intensive (Hartnell et al., 2019). Moreover, the culture-based methods are not sensitive to the viable but non-culturable (VBNC) status of the bacterium, and other bacteria with similar biochemical characteristics interfere (O’Hara et al., 2003; Li et al., 2014). Polymerase chain reaction (PCR)- and quantitative PCR (qPCR)-based methods have been developed for detecting *V. vulnificus* and have showed high sensitivity and specificity (Panicker and Bej, 2005; Kumar et al., 2006; D’Souza et al., 2019). However, these methods require sophisticated thermal-cycling equipment and other laboratory settings, and the usage is limited. Loop-mediated isothermal amplification (LAMP), a nucleic acid isothermal amplification technology, has been applied to develop simpler assays for the detection of *V. vulnificus* (Han and Ge, 2010; Surasilp et al., 2011).

Recombinase polymerase amplification (RPA), another isothermal amplification technology, has been widely applied for foodborne pathogen detections in recent years for its rapidness, simplicity, and convenience (Piepenburg et al., 2006; Du et al., 2018; Dong et al., 2020). The amplification products of RPA can be analyzed by gel electrophoresis, lateral flow chromatography, or fluorescence (Li et al., 2018). Among them, the fluorescence analysis makes real-time reading of the signal possible, and this “real-time RPA” assay has a good combination of speed, portability, and accessibility (Piepenburg et al., 2006). Briefly, the real-time RPA reaction contains an “exo probe” that anneals to one of the amplified strands and recruits an exonuclease to cleave off the tetrahydrofuran (THF) substitution on the probe to expose the 3’ end for strand extension. The cleavage also separates the fluorophore and the quenching group at the two sides of the THF on the probe, emitting a fluorescence signal (Piepenburg et al., 2006).

In this study, a real-time RPA method for rapid detection of *V. vulnificus* has been established. This method finishes the detection in 14 min with good specificity. The limit of detection was 17 gene copies or 1 colony-forming unit (CFU) per reaction, or 1 CFU/10 g of spiked food with enrichment. Detection results were 100% consistent with bioassay and qPCR for clinical food samples. Moreover, the method could resist the effect of food matrix and could tolerate crude templates, making it a well portable method to be widely applied for *V. vulnificus* detection in food safety control.

## Materials and Methods

### Bacteria Strains

A collection eight *Vibrio vulnificus* environmental strains and reference strains of *Vibrio cholerae*, *Vibrio harveyi*, *Vibrio mediterranei*, and *Vibrio shilonii* were obtained from Jiangsu Institute of Oceanology and Marine Fisheries (Nantong, China). Reference strains of *V*. *vulnificus*, *Vibrio parahaemolyticus*, *Vibrio alginolyticus*, *Salmonella* Typhimurium, *Listeria monocytogenes*, *Bacillus cereus*, and *Staphylococcus aureus* were kindly provided by the Wuhan Institute for Food and Cosmetic Control (Wuhan, China). Reference strains of *Vibrio splendidus*, *Vibrio ichthyoenteri*, *Vibrio mimicus*, *Vibrio campbellii*, *Vibrio chagasii*, *Vibrio fluvialis*, *Vibrio natriegens*, *Vibrio* sp., *Vibrio ponticus*, *Vibrio rotiferianus*, and *Vibrio diabolicus* were purchased from the Ministry of Natural Resources Third Institute of Oceanography (Xiamen, China). All the bacterial strains were confirmed by 16S rRNA sequencing (Hiergeist et al., 2016). The details of the strains are listed in Table 1.

**TABLE 1:** Information of bacteria strains used in this study.

**Species**	**Source**	**Origin/designation**	**Real-time RPA**
*Vibrio vulnificus*	Reference strain	ATCC 29307	+ (positive)
*V.vulnificus*	Environmental strain	Nantong, China	+
*V.vulnificus*	Environmental strain	Nantong, China	+
*V.vulnificus*	Environmental strain	Nantong, China	+
*V.vulnificus*	Environmental strain	Nantong, China	+
*V.vulnificus*	Environmental strain	Wuhan, China	+
*V.vulnificus*	Environmental strain	Wuhan, China	+
*V.vulnificus*	Environmental strain	Lianyungang, China	+
*V.vulnificus*	Environmental strain	Lianyungang, China	+
*Vibrio parahaemolyticus*	Reference strain	ATCC 17802	− (negative)
*Vibrio alginolyticus*	Reference strain	ATCC 17749	−
*Salmonella* Typhimurium	Reference strain	ATCC 14028	−
*Listeria monocytogenes*	Reference strain	ATCC 19115	−
*Bacillus cereus*	Reference strain	ATCC 14579	−
*Staphylococcus aureus*	Reference strain	ATCC 6538	−
*Vibrio cholerae*	Reference strain	ATCC 14100	−
*Vibrio harveyi*	Reference strain	ATCC 43516	−
*Vibrio mediterranei*	Reference strain	ATCC 43341	−
*Vibrio shilonii*	Reference strain	ATCC BAA-91	−
*Vibrio splendidus*	Reference strain	MCCC 1A04096	−
*Vibrio mimicus*	Reference strain	MCCC1A02602	−
*Vibrio ichthyoenteri*	Reference strain	MCCC1A00057	−
*Vibrio campbellii*	Reference strain	MCCC 1A02605	−
*Vibrio* sp.	Reference strain	MCCC 1A00047	−
*Vibrio chagasii*	Reference strain	MCCC 1B00386	−
*Vibrio fluvialis*	Reference strain	MCCC 1A02761	−
*Vibrio natriegens*	Reference strain	MCCC 1D00129	−
*Vibrio ponticus*	Reference strain	MCCC 1H00061	−
*Vibrio rotiferianus*	Reference strain	MCCC 1B00068	−
*Vibrio diabolicus*	Reference strain	MCCC 1D00126	−

*RPA, recombinase polymerase amplification.*

### Sequence Analysis

Multiple sequence alignments were performed on *empV* and *gyr*B gene sequences of all *V. vulnificus* and other selected *Vibrio* species available in GenBank using Clustal^1^ with the default parameters; then the diverged sequences flanking the conservative core were ignored. A phylogenetic tree was constructed based on the alignments using MEGA4^2^ with the neighbor-joining (NJ) method.

### Primer/Probe Design

For design of primers, the FASTA sequence of *empV* gene (GenBank No. U50548.1) was input into the National Center for Biotechnology Information (NCBI) Primer-BLAST^3^. The primer BLAST was performed with the following criteria put into consideration: (1) the primer pair should only target the species of interest (*V. vulnificus*); (2) the primer pair should have less than four consecutive bases (and less than one if located at the 3’ end) pairing each other. The product size was set as minimum at 150 and maximum at 500. The database was set as Refseq representative genomes. The organism was set as *V. vulnificus* (taxid: 672). The primer size was set as minimum at 28 and maximum at 35. The primer guanine–cytosine (GC) content was set as minimum at 20 and maximum at 80. The maximal self-complementarity was set as any at 4 and 3’ at 1. The maximal pair complementarity was set as any at 4 and 3’ at 1. Other parameters were set as default. For the probe design, the sequence of region defined by the primer pair was input into the Primer Premier 5 software. The size of the probe was set as minimum at 46 and maximum at 52. The melting temperature (*Tm*) was set as minimum at 50 and maximum at 100. The GC content was set as minimum at 20 and maximum at 70. The maximum hairpin score was set as 9. The maximum primer-dimer score was set as 9. The maximum poly-X was set as 5. Other parameters were set as default. The probe had a C3 spacer (SpC3) at the 3’ end, which could block strand extension, and a THF group at the middle (position 32) to facilitate exonuclease III (exo) cutting. Only when the bases flanking the THF site of the probe had a good pairing with the template would the exo cutting occur to release the 3’ end from SpC3 blocking. Moreover, two T bases on the both sites of THF site were substituted by FAM (6-corboxy-fluorescein)-dT and BHQ1 (Black Hole Quencher 1)-dT. Primers and probe were synthesized by General Biosystems Co., Ltd., Anhui, China.

### Real-Time Recombinase Polymerase Amplification Procedure

If bacterial cultures were used as the templates, they were diluted to the desired concentrations if necessary, treated at 100°C for 10 min and immediately used. For reactions using purified genomic DNA as the templates, the genomic DNA was extracted using TIANamp Genomic DNA Kit (Tiangen Biotech Co., Ltd., Beijing, China) and quantified using Qubit 4 (Thermo Fisher Scientific Inc., Wilmington, DE, United States). The copy number of targeted DNA fragment was calculated based on the genome size (4.97039 Mb). Real-time RPA reactions were set up according to the manufacturer’s instructions of TwistAmp DNA Amplification exo Kit (TwistDx Inc., Maidenhead, United Kingdom). The reaction contained 29.5 μl of rehydration buffer, 2.1 μl of each primer (10 μM), 0.6 μl of probe (10 μM), 12.2 μl of distilled water, 1 μl of the template, and a dried enzyme pellet. To initiate the reaction, 2.5 μl of magnesium acetate (280 mM) was added to the mixture. After a brief centrifugation, the reaction mixture was pre-incubated at 39°C for 4 min. Subsequently, the fluorescence signal was recorded in real-time on a Roche LightCycler 480 II qPCR machine at 39°C in the FAM channel, with signal reading at 13-s intervals for 25 min.

### Preparation of Spiked Food Samples

Shrimp, oyster, fish flesh (pomfret), and crab were purchased from a local market and verified to be free of *V. vulnificus* by quantitative PCR (Kumar et al., 2006). Food samples were homogenized thoroughly using a handheld grinder (3rd Gen. TGrinder, Tiangen Biotech Co., Ltd.). Ten grams of the food homogenate was made to 100 ml with alkaline peptone broth (Sinopharm Chemical Reagent Co., Ltd., Beijing, China) and spiked with desired amounts of *V. vulnificus*. If no enrichment was needed, 1 ml of the solution was boiled at 100°C for 10 min and centrifuged at 5,000 *g* for 5 min. One microliter of the supernatant was used as the template for real-time RPA detection. If enrichment was needed, the spiked food samples were incubated at 30°C with 200-rpm shaking. A total of 1 ml of the enrichment solution was collected at different time points and centrifuged at 800 *g* for 10 min to remove food debris. Bacterial cells were pelleted with 5,000 *g* centrifugation for 5 min, resuspended with 200 μl of water, and boiled at 100°C for 10 min. Also, 1 μl of the boiled resuspension was used as the template for real-time RPA detection.

### Clinical Samples

The clinical seafood samples (shrimp, fish, shellfish, and crab) were kindly provided by the Jiangsu Institute of Oceanology and Marine Fisheries (Nantong, China). After a disinfection treatment with ethanol, 9 ml of phosphate-buffered saline (PBS) was added to 1 g of each sample and homogenized thoroughly using the handheld grinder. Then 1 ml of the sample homogenate was boiled at 100°C for 10 min and centrifuged at 800 *g* for 10 min to remove food debris. A total of 1 μl of the supernatant was used as template for real-time RPA or quantitative PCR detection. For the bioassay, the procedure followed the National Standard of China DBS13/004-2016. Briefly, 10 g of the clinical seafood samples was added into 90 ml of alkaline saline peptone water and incubated at 37°C for 18–24 h. The cultures were line separated on mCPC medium plates at 37°C overnight. Suspected single colonies were inoculated onto Luria broth (LB) agar plates with 3% NaCl and incubated at 37°C for 18–24 h. Then oxidase test, gram stain, halophilic test, and ONPG test were performed.

### Quantitative PCR

The qPCR detection of *V. vulnificus* followed an established method reported previously (Kumar et al., 2006). Specifically, the primer pair targeting the *gyr*B gene was used (Table 2). The qPCR mixture contained 25 μl of the 2 × SYBR Green qPCR Mix (Tiangen Biotech Co., Ltd.), 0.4 μl of each primer (10 μM), and 1 μl of the template. The cycling program was 95°C 10 min followed by 45 cycles of 95°C for 20 s, 55°C for 20 s, and 72°C for 30 s on a Roche LightCycler 480 II qPCR machine. The melting curve analysis was set as default. Cycle threshold (*Ct*) values less than 32 were considered as positive.

**TABLE 2:** Primer and probe sequences.

**No.**	**Name**	**Sequence (5′–3′)**	**Length (bp)**	**Amplicon size (bp)**	**Amplification target on gene**	**GenBank no. of gene**
1	F (forward primer)	5′-GAGATGGATTCTTTGTATAACATTGCGT-3′	28	214	539.752	U50548.1
	R (reverse primer)	5′-ACGATGACGTTGGTTGTGTTTCATTATC-3′	28			(*empV*)
	P (probe)	5′-GAAGTTGGCTGGTGGTTATTTTCTGAACCA [FAM-dT][THF]GT[BHQ1-dT]GTTGAGCTCGCAG-3′	47			
2 (Kumar et al., 2006)	F (forward primer)	5′-GTCCGCAGTGGAATCCTTCA-3′	20	286	1041.1326	BA000037.2
	R (reverse primer)	5′-TGGTTCTTACGGTTACGGCC-3′	20			(*gyr*B)

### Statistical Analysis

For the limit of detection data of the real-time RPA assay, a probit regression analysis was performed using SPSS software (IBM, Armonk, NY, United States), and a semi-log regression analysis was carried out using GraphPad Prism 8.0 (GraphPad Software Inc., San Diego, CA). For the clinical sample detection data, the correlation between threshold time (*Tt*) value of real-time RPA and cycle time (*Ct*) value of qPCR was analyzed using GraphPad Prism 8.0.

## Results

### Selection of the Target Gene and Primer/Probe Design

**FIGURE 1:**
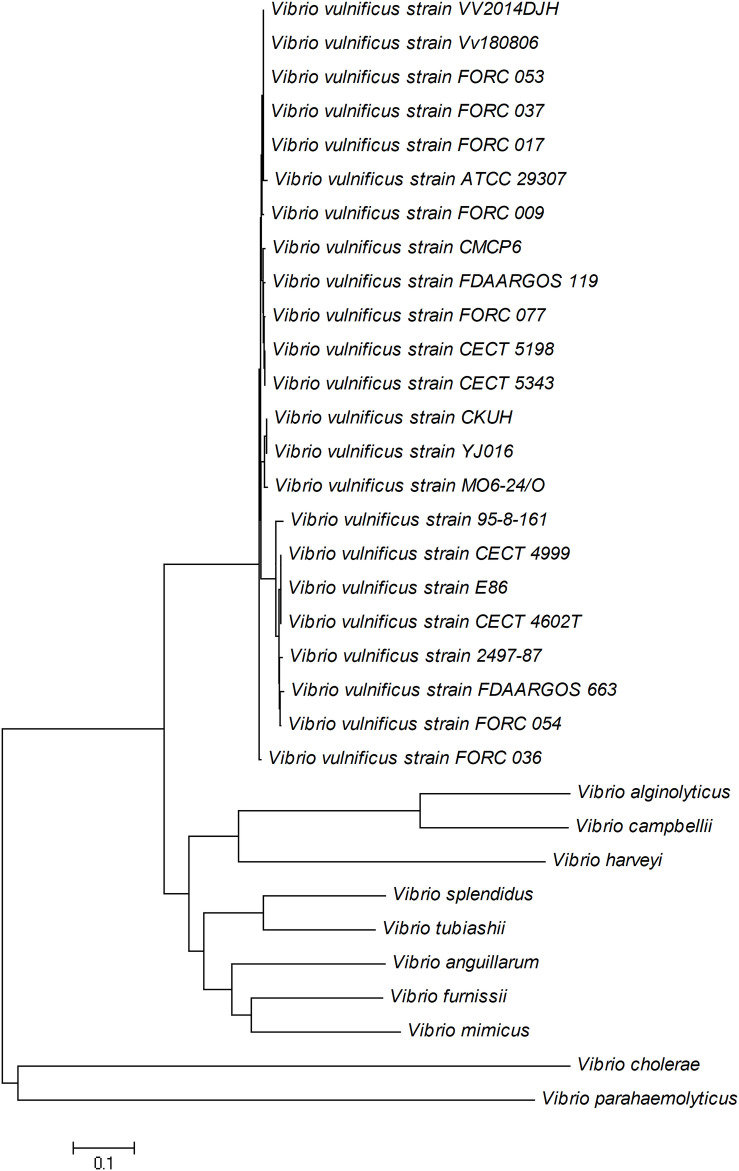
Neighbor-joining tree of *empV* gene of 23 *Vibrio vulnificus* strains and 10 other *Vibrio* species. GenBank accession numbers: *V. vulnificus* CMCP6 (CP037932.1), *V. vulnificus* 95-8-161 (AB540652.1), *V. vulnificus* 2497-87 (CP060048.1), *V. vulnificus* CECT 4602T (AM492792.1), *V. vulnificus* CECT 4999 (CP014637.1), *V. vulnificus* CECT 5198 (AB540651.1), *V. vulnificus* CECT 5343 (AB540651.1), *V. vulnificus* CKUH (AB540650.1), *V. vulnificus* E86 (DQ923325.1), *V. vulnificus* Env1 (CP017636.1), *V. vulnificus* FDAARGOS_119 (CP014048.2), *V. vulnificus* FDAARGOS_663 (CP044068.1), *V. vulnificus* FORC_009 (CP009985.1), *V. vulnificus* FORC_017 (CP012740.1), *V. vulnificus* FORC_036 (CP015513.1), *V. vulnificus* FORC_037 (CP016322.1), *V. vulnificus* FORC_053 (CP019291.1), *V. vulnificus* FORC_054 (CP019122.1), *V. vulnificus* FORC_077 (CP027031.1), *V. vulnificus* MO6-24/O (CP002470.1), *V. vulnificus* VV2014DJH (CP019321.1), *V. vulnificus* Vv180806 (CP044207.1), *V. vulnificus* YJ016 (BA000038.2), *Vibrio alginolyticus* (NZ_CP042449.1), *Vibrio anguillarum* (NZ_AEZA01000040.1), *Vibrio campbellii* (NZ_CP019634.1), *Vibrio cholerae* (NZ_AP014524.1), *Vibrio furnissii* (NZ_CP040990.1), *Vibrio harveyi* (NZ_CP009467.1), *Vibrio mimicus* (NZ_ADAF01000001.1), *Vibrio splendidus* (NZ_CP031055.1), *Vibrio tubiashii* (CP009354.1), and *Vibrio parahaemolyticus* (CP012950.1).

The conservative gene *empV* encoding the extracellular metalloproteinase of *Vibrio vulnificus* was selected as the detection target of the real-time RPA method (Chuang et al., 1997). The evolutionary conservation of the *empV* gene was confirmed by construction of an unrooted NJ phylogenetic tree based on multiple sequence alignments of *empV* gene sequences of 23 *V. vulnificus* strains and 10 other selected *Vibrio* species. All the 23 *empV* gene sequences of *V. vulnificus* strains were classified into one group that was a distant relative of the other *Vibrio* species (Figure 1). This result indicated that the *empV* gene had good specificity and evolutionary conservation. The NCBI Primer-BLAST search for primer candidates on the sequence of gene *mepV* returned with five potential primer pairs. These primers were tentatively screened by amplification of the target gene fragment with the no-template control. The amplification products were electrophorized on agarose gel to compare amplification performance of the target and primer-dimer formation in the no-template control. The primer pair showing the best amplification performance without a sign of primer-dimer formation was selected, and a probe was designed for this primer pair. The sequences of this primer-probe set are listed in Table 2.

### Specificity of the Method

To evaluate the specificity of the real-time RPA method, culture solutions of a number of bacterial strains, including environment isolates of *V. vulnificus*, other *Vibrio* species, and a number of other foodborne pathogenic bacteria, at a cell density of 10^6^ CFU/ml, were tested (Table 1). The other *Vibrio* species and other foodborne pathogenic bacteria were all negative, and the *V. vulnificus* reference strain and environmental isolates were all detected. This result confirmed the specificity of the real-time RPA method.

### Limit of Detection of the Method

**FIGURE 2:**
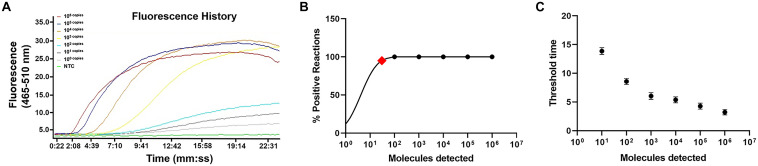
Limit of detection of real-time recombinase polymerase amplification (RPA) method. **(A)** The fluorescence history diagram of the results of real-time RPA with different amounts (in copies) of *Vibrio vulnificus*. The amounts tested are indicated with different colors. NTC, no-template control. The diagram was one typical outcome of eight independent experiments. **(B)** Probit regression analysis of the data collected from the eight real-time RPA repeats using SPSS software. The limit of detection at 95% probability (17 copies/reaction) is depicted by a red rhomboid. **(C)** Semi-logarithmic regression of the data collected from the eight real-time RPA repeats using GraphPad Prism 8.0. The run time of the real-time RPA was ∼2–14 min for the templates at 10^6^–10^0^ copies/reaction.

**FIGURE 3:**
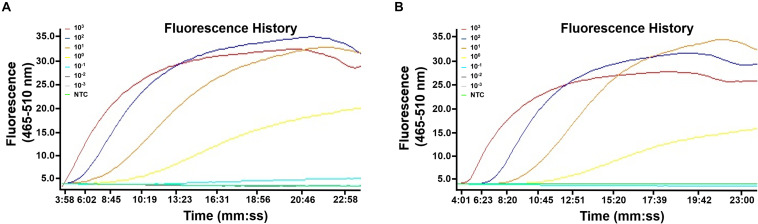
Limit of detection of the real-time recombinase polymerase amplification (RPA) method. The fluorescence history diagrams show the results of real-time RPA with different amounts (in CFU) of *Vibrio vulnificus*. The amounts tested are indicated with different colors. Templates from culture solution **(A)** or spiked shrimp samples **(B)** were tested. NTC, no-template control. The diagrams are one typical outcome of three independent experiments.

The limit of detection of the real-time RPA method was evaluated under various conditions. Firstly, the purified genomic DNA of *V. vulnificus* with the concentration of 10^0^–10^6^ copies per microliter was tested (1 μl for each reaction) (Figure 2A). The real-time RPA assay was performed for eight independent repeats; 10^2^ copies and above were detected in 8/8 runs, 10^1^ copies were detected in 7/8 runs, and 10^0^ copies were detected in 2/8 runs. A probit regression analysis was carried out for the data of the 8 repeats, and the result showed that the limit of detection was 17 copies/reaction in 95% of cases (Figure 2B). Semi-log regression analysis of the data of the 8 repeats showed that the reaction time lengths of the real-time RPA assay were 2–14 min for 10^6^–10^1^ copies (Figure 2C). Secondly, a 10-fold series dilution of *V. vulnificus* culture ranging from 10^6^ to 10^0^ CFU/ml (10^3^–10^–3^ CFU per reaction) was tested. Signal of 10^0^ CFU could be observed (Figure 3A). This limit of detection was not affected by the food matrix (shrimp homogenate) (Figure 3B). Thirdly, the limit of detection was tested in spiked food samples with different enrichment time. Ten grams of shrimp, oyster, fish flesh, and crab homogenate was spiked with 10^0^–10^3^ CFU of *V. vulnificus* and enriched for 0–24 h. The results showed that 1 CFU/10 g could be detected after 4 h of enrichment for all the four food matrix types (Table 3). Thus, the limit of detection of the real-time RPA method was 17 copies or 1 CFU per reaction, or 1 CFU/10 g in spiked food samples with 4 h of enrichment.

**TABLE 3:** Detection of *Vibrio vulnificus* in spiked food samples.

**Food sample**	**Spike concentration (CFU/10 g)**	**Result of real-time RPA**						
		**0 h**	**2 h**	**4 h**	**6 h**	**8 h**	**12 h**	**24 h**
shrimp	10^0^	−	−	+	+	+	+	+
	10^1^	−	−	+	+	+	+	+
	10^2^	−	+	+	+	+	+	+
	10^3^	+	+	+	+	+	+	+
Fish	10^0^	−	−	+	+	+	+	+
	10^1^	−	−	+	+	+	+	+
	10^2^	−	+	+	+	+	+	+
	10^3^	+	+	+	+	+	+	+
Crab	10^0^	−	−	+	+	+	+	+
	10^1^	−	−	+	+	+	+	+
	10^2^	−	+	+	+	+	+	+
	10^3^	+	+	+	+	+	+	+
Oyster	10^0^	−	−	+	+	+	+	+
	10^1^	−	−	+	+	+	+	+
	10^2^	−	+	+	+	+	+	+
	10^3^	+	+	+	+	+	+	+

*+, positive result; −, negative result. RPA, recombinase polymerase amplification.*

### Clinical Sample Detection

**FIGURE 4:**
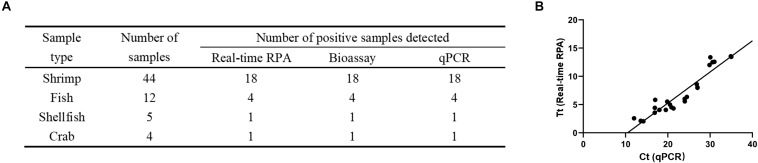
Detection of *Vibrio vulnificus* in clinical samples. **(A)** The detection of *V. vulnificus* for each clinical sample was performed by real-time recombinase polymerase amplification (RPA), bioassay, and qPCR. **(B)** Linear regression analysis of real-time RPA threshold time (*Tt*) and qPCR cycle threshold values (*Ct*) using GraphPad Prism software. *R*^2^ = 0.8936.

A total of 65 clinical seafood samples including 44 shrimp, 12 fish, 5 shellfish, and 4 crab samples were tested for *V. vulnificus* with real-time RPA, culturing-based bioassay (National Standard of China DBS13/004-2016), and qPCR. The detection results of real-time RPA were consistent with the bioassay and qPCR results (Figure 4A and Supplementary Table S1). Moreover, the threshold time (*Tt* value) of real-time RPA ranged from 2.05 to 14.23 min, while the cycle threshold (*Ct* value) of qPCR ranged from 13.58 to 35 cycles. The correlation of the *Ct* value and *Tt* value of the 24 positive samples was analyzed. The result showed that a well correlation was observed with an R^2^ value of 0.8936 (Figure 4B).

## Discussion

*Vibrio vulnificus* is a prevalent foodborne pathogen that leads to significant economic losses and public health concern (Jones and Oliver, 2009). Rapid and accurate detection method of *V. vulnificus* in food can help control the related epidemics and diseases. This study described the development and evaluation of a real-time RPA method for detecting *V. vulnificus*. The real-time RPA method finishes in 14 min with good specificity and sensitivity and has satisfactory tolerance for crude samples. Being rapid and simple, the method is potentially applied in a wide range of conditions.

The method targets the *empV* gene encoding the extracellular metalloprotease. This gene has a highly conservative property (Chuang et al., 1997), and the phylogenetic analysis showed that all the *empV* gene sequences of 23 *V. vulnificus* strains were classified into one group while other *Vibrio* species were classified into other groups, predicting good detection coverage and specificity. The *gyr*B gene has also been utilized as a target for detection of *V. vulnificus* (Kumar et al., 2006; D’Souza et al., 2019). Just as in the case of *empV*, the phylogenetic tree of *gyr*B gene sequences also predicted good detection coverage and specificity, indicating equivalent evolutionary conservation of the two genes (Supplementary Figure S1). The results in this study showed excellent specificity to *V. vulnificus* and good coverage for the isolated environment strains. Moreover, in the clinical sample tests, results of real-time RPA were consistent with those of qPCR (targeting *gyr*B gene) and culture-based bioassay. Therefore, the *empV* gene is an effective detection target.

The real-time RPA method showed good detection sensitivity that was comparable with that of qPCR and LAMP. The limit of detection was 17 copies/reaction in 95% of the cases or 1 CFU per reaction, or 1 CFU/10 g in spiked food samples with enrichment. In other reports, the limit of detection of *V. vulnificus* was 0.1–1 CFU/reaction with qPCR-based methods or 10–100 CFU/reaction with LAMP (Panicker and Bej, 2005; Wang et al., 2016; D’Souza et al., 2019). The limit of detection of the real-time RPA method was also comparable with that of other RPA-based methods for detection of other pathogens, such as ∼10 CFU per reaction (*Vibrio harveyi* and *Listeria monocytogenes*) or 50 copies per reaction (*Vibrio parahaemolyticus*) (Garrido-Maestu et al., 2019; Pang et al., 2019; Yang et al., 2020).

As reported, the RPA reaction has a good tolerance for crude samples (Moore and Jaykus, 2017). This is also true for the real-time RPA method of this study. In our culture, spiked food, and clinical samples, DNA was released by simple boiling and directly used for the detection, and the limit of detection and accuracy were not affected. This makes the overall procedure of detection even simpler. Although we used a qPCR machine to read the fluorescence signal in this study, a portable tube scanner (such as Genie III from Beijing Suntrap Science & Technology Co., Ltd.) could also give satisfactory results (Geng et al., 2019). Thus, the real-time RPA method is composed of two rapid and simple steps: sample boiling and isothermal amplification with real-time signal reading, which are very portable and can be applied widely.

In conclusion, a real-time RPA method was developed for rapid detection of *V. vulnificus*. It is an efficient and reliable detection tool for *V. vulnificus* in the food safety control.

## Data Availability Statement

The raw data supporting the conclusions of this article will be made available by the authors, without undue reservation, to any qualified researcher.

## Author Contributions

JD directed the program. SG, JD, and XY designed the research. XY and XZ performed the experiments. HS, GJ, and YW processed the data. XY and PZ wrote the manuscript. All authors contributed to the article and approved the submitted version.

## Conflict of Interest

The authors declare that the research was conducted in the absence of any commercial or financial relationships that could be construed as a potential conflict of interest.

## Footnotes

1 http://www.clustal.org/

2 http://www.megasoftware.net/mega4/mega.html

3 https://www.ncbi.nlm.nih.gov/tools/primer-blast
