# Supplementary material for: A Real-Time Recombinase Polymerase Amplification Method for Rapid Detection of Vibrio vulnificus in Seafood
Source: Front Microbiol. 2020 Nov 6;11:586981. doi: 10.3389/fmicb.2020.586981 (PMC7677453; doi:10.3389/fmicb.2020.586981)
Supplement: Supplementary file 1 [file Data_Sheet_1.PDF]

Supplementary File

A real-time recombinase polymerase amplification method for rapid detection of *Vibrio vulnificus* in seafood

Xiaohan Yang[1,†], Xue Zhang[1,†], Yu Wang[1], Hui Shen[2], Ge Jiang[2], Jingquan Dong[1,*], Panpan Zhao[3,*], Song Gao[1,*]

[1]Jiangsu Key Laboratory of Marine Biological Resources and Environment, Jiangsu Key Laboratory of Marine Pharmaceutical Compound Screening, Co-Innovation Center of Jiangsu Marine Bio-industry Technology, School of Pharmacy, Jiangsu Ocean University, Lianyungang 222005, China

[2]Jiangsu Institute of Oceanology and Marine Fisheries, Nantong 226007, China

[3]Key Laboratory of Zoonosis Research by Ministry of Education, College of Veterinary Medicine, Jilin University, Changchun 130062, China

[†]These authors contributed equally to this paper.

*Correspondence:

Jingquan Dong 2018000029@jou.edu.cn

Panpan Zhao zhaopp19@mails.jlu.edu.cn

Song Gao gaos@jou.edu.cn

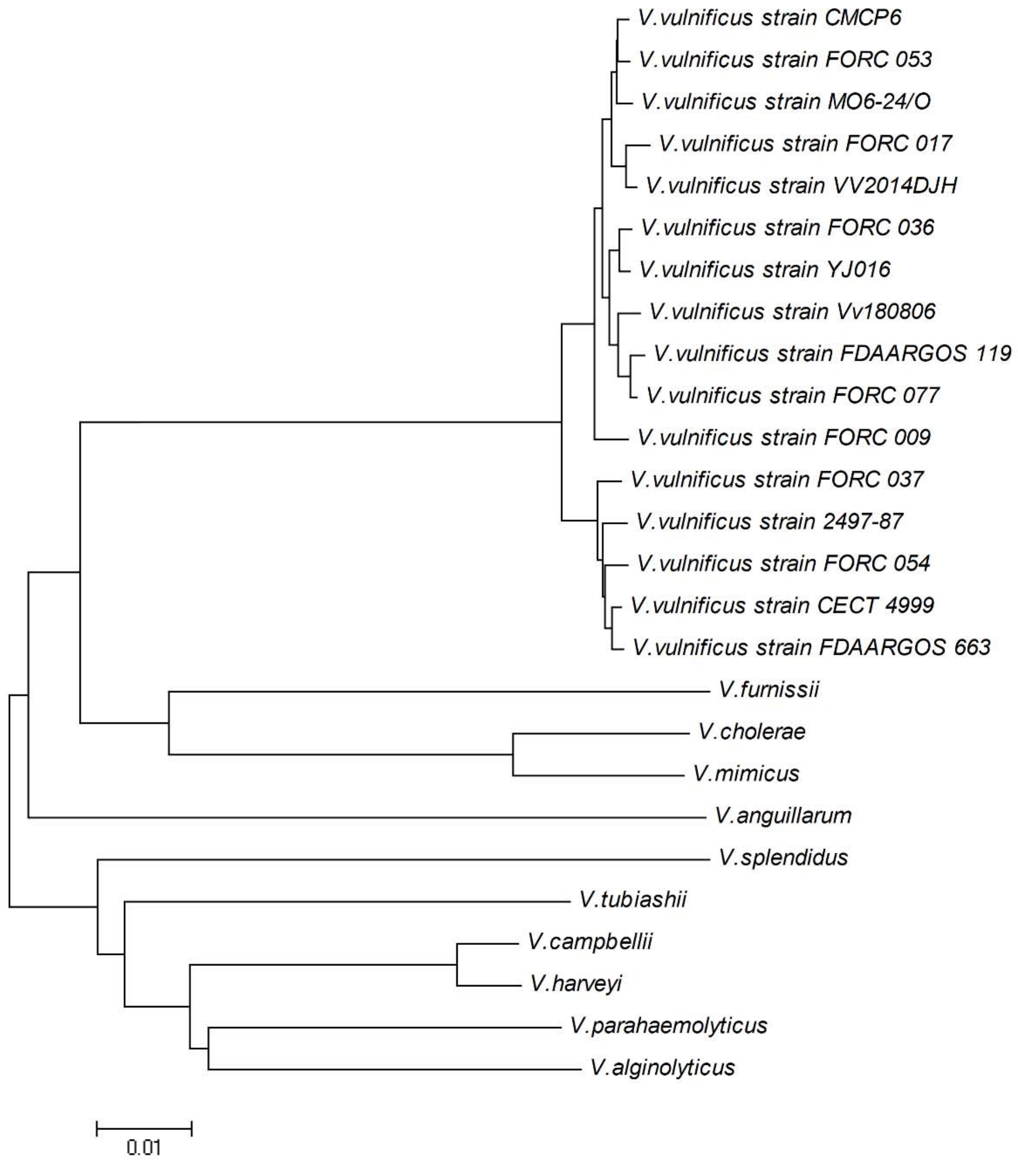

Figure S1 Neighbor-joining tree of *gyr*B gene of *V. vulnificus* strains and other *vibrio* species. GenBank accession numbers: *V. vulnificus* CMCP6 (CP037931.1), *V. vulnificus* 2497-87 (CP060047.1), *V. vulnificus* CECT 4999 (CP014636.1), *V. vulnificus* FDAARGOS_119 (CP014049.2), *V. vulnificus* FDAARGOS_663 (CP044069.1), *V. vulnificus* FORC_009 (CP009984.1), *V. vulnificus* FORC_017 (CP012739.1), *V. vulnificus* FORC_036 (CP015512.1), *V. vulnificus* FORC_037 (CP016321.1), *V. vulnificus* FORC_053 (CP019290.1), *V. vulnificus* FORC_054 (CP019121.1), *V. vulnificus* FORC_077 (CP027030.1), *V. vulnificus* MO6-24/O (CP002469.1), *V. vulnificus* VV2014DJH (CP019320.1), *V. vulnificus* Vv180806 (CP044206.1), *V. vulnificus* YJ016 (BA000037.2), *V. alginolyticus* (AP022866.1), *V. anguillarum* (NZ_AEZA01000061.1), *V. campbellii* (CP033135.1), *V. cholerae* (CP053797.1), *V. furnissii* (MG954380.1), *V. harveyi* (AY630354.1), *V. mimicus* (AB435238.1), *V. splendidus* (DQ987707.1), *V. tubiashii* (CP009355.1), *V. parahaemolyticus* (DQ479431.1).

Table S1 Detection of *V. vulnificus* in clinical samples.

No.	Food type	Species	Tissue	Detection results		
				Real-time RPA	Bioassay	qPCR
1	Shrimp	*Exopalamon carincauda*	hepatopancreas	+	+	+
2	Shrimp	*Exopalamon carincauda*	hepatopancreas	-	-	-
3	Shrimp	*Exopalamon carincauda*	hepatopancreas	-	-	-
4	Shrimp	*Exopalamon carincauda*	hepatopancreas	+	+	+
5	Shrimp	*Exopalamon carincauda*	gill	-	-	-
6	Shrimp	*Exopalamon carincauda*	gill	-	-	-
7	Shrimp	*Exopalamon carincauda*	gill	+	+	+
8	Shrimp	*Exopalamon carincauda*	gill	+	+	+
9	Shrimp	*Litopenaeus vannamei*	hepatopancreas	-	-	-
10	Shrimp	*Litopenaeus vannamei*	hepatopancreas	-	-	-
11	Shrimp	*Litopenaeus vannamei*	hepatopancreas	-	-	-
12	Shrimp	*Litopenaeus vannamei*	hepatopancreas	-	-	-
13	Shrimp	*Litopenaeus vannamei*	hepatopancreas	-	-	-
14	Shrimp	*Litopenaeus vannamei*	hepatopancreas	+	+	+
15	Shrimp	*Litopenaeus vannamei*	hepatopancreas	-	-	-
16	Shrimp	*Litopenaeus vannamei*	hepatopancreas	-	-	-
17	Shrimp	*Litopenaeus vannamei*	hepatopancreas	+	+	+
18	Shrimp	*Litopenaeus vannamei*	hepatopancreas	-	-	-
19	Shrimp	*Litopenaeus vannamei*	hepatopancreas	-	-	-
20	Shrimp	*Litopenaeus vannamei*	hepatopancreas	-	-	-
21	Shrimp	*Litopenaeus vannamei*	hepatopancreas	+	+	+
22	Shrimp	*Litopenaeus vannamei*	hepatopancreas	-	-	-
23	Shrimp	*Litopenaeus vannamei*	hepatopancreas	-	-	-
24	Shrimp	*Litopenaeus vannamei*	hepatopancreas	+	+	+
25	Shrimp	*Litopenaeus vannamei*	hepatopancreas	+	+	+
26	Shrimp	*Litopenaeus vannamei*	hepatopancreas	-	-	-
27	Shrimp	*Litopenaeus vannamei*	hepatopancreas	+	+	+
28	Shrimp	*Litopenaeus vannamei*	hepatopancreas	-	-	-
29	Shrimp	*Litopenaeus vannamei*	hepatopancreas	+	+	+
30	Shrimp	*Litopenaeus vannamei*	hepatopancreas	-	-	-
31	Shrimp	*Litopenaeus vannamei*	hepatopancreas	-	-	-
32	Shrimp	*Litopenaeus vannamei*	hepatopancreas	+	+	+
33	Shrimp	*Litopenaeus vannamei*	hepatopancreas	+	+	+
34	Shrimp	*Litopenaeus vannamei*	hepatopancreas	+	+	+
35	Shrimp	*Litopenaeus vannamei*	hepatopancreas	-	-	-
36	Shrimp	*Litopenaeus vannamei*	intestinal	-	-	-
37	Shrimp	*Litopenaeus vannamei*	intestinal	-	-	-
38	Shrimp	*Litopenaeus vannamei*	intestinal	+	+	+
39	Shrimp	*Litopenaeus vannamei*	intestinal	+	+	+
40	Shrimp	*Litopenaeus vannamei*	gill	-	-	-
41	Shrimp	*Litopenaeus vannamei*	gill	+	+	+
42	Shrimp	*Litopenaeus vannamei*	gill	-	-	-

(Table continued in the next page.)

Table S1 (continued)

No.	Food type	Species	Tissue	Detection results		
				Real-time RPA	Bioassay	qPCR
43	Shrimp	*Litopenaeus vannamei*	gill	-	-	-
44	Shrimp	*Litopenaeus vannamei*	gill	-	-	-
45	Fish	*Monotaxis grandoculis*	gill	+	+	+
46	Fish	*Monotaxis grandoculis*	gill	-	-	-
47	Fish	*Monotaxis grandoculis*	gill	-	-	-
48	Fish	*Monotaxis grandoculis*	gill	-	-	-
49	Fish	*Monotaxis grandoculis*	gill	-	-	-
50	Fish	*Mugil cephalus*	gill	-	-	-
51	Fish	*Mugil cephalus*	gill	+	+	+
52	Fish	*Mugil cephalus*	gill	+	+	+
53	Fish	*Carassius auratus*	liver	-	-	-
54	Fish	*Carassius auratus*	liver	-	-	-
55	Fish	*Carassius auratus*	liver	-	-	-
56	Fish	*Carassius auratus*	liver	+	+	+
57	Shellfish	*ostrea gigas thunberg*	meat	-	-	-
58	Shellfish	*ostrea gigas thunberg*	meat	-	-	-
59	Shellfish	*ostrea gigas thunberg*	meat	-	-	-
60	Shellfish	*ostrea gigas thunberg*	meat	-	-	-
61	Shellfish	*ostrea gigas thunberg*	meat	+	+	+
62	Crab	*Eriocheir sinensis*	gill	-	-	-
63	Crab	*Eriocheir sinensis*	gill	-	-	-
64	Crab	*Eriocheir sinensis*	gill	-	-	-
65	Crab	*Eriocheir sinensis*	gill	+	+	+

(+: positive result; -: negative result.)
